# Dispatcher referral of bystanders to retrieve drone-delivered automated external defibrillators in cases of suspected out-of-hospital cardiac arrest

**DOI:** 10.1016/j.resplu.2026.101262

**Published:** 2026-02-16

**Authors:** Sofia Schierbeck, Anette Nord, Leif Svensson, Magnus Kristiansson, Mattias Ringh, Per Nordberg, Jacob Hollenberg, Gabriel Riva, Martin Jonsson, Andreas Claesson

**Affiliations:** a Centre for Resuscitation Science, Department of Clinical Science and Education, Södersjukhuset, Karolinska Institutet, Stockholm, Sweden; b Department of Physiology and Pharmacology, Karolinska Institutet, Stockholm, Sweden; c Function Perioperative Medicine and Intensive Care, Section for Cardiothoracic Anaesthesia and Intensive Care, Karolinska University Hospital, Stockholm, Sweden

**Keywords:** AED, Drone, OHCA, Referral, UAV, EMDC

## Abstract

**Background:**

Drone dispatch in out-of-hospital cardiac arrest (OHCA) may shorten time to AED-attachment, however little is known about barriers for dispatchers to refer bystanders to retrieve and attach AEDs after delivery. This study aims to evaluate characteristics of drone delivered AEDs (D-AEDs) before and after implementing an educational bundle.

**Methods:**

This retrospective before–after observational study included all cases of D-AEDs arriving before EMS in suspected OHCA in Sweden (2020–2023). A supportive educational bundle was implemented at the dispatch centre in June 2022. Dispatcher–caller interactions were evaluated through audits of 112 voice logs using a modified CARES protocol.

**Results:**

Out of 123 deliveries of D-AEDs to suspected OHCAs before EMS arrival, 62 cases (50%) received bystander CPR. Dispatcher referral of bystanders to retrieve the D-AED occurred in totally 30/62 (48%) cases with an increase from 7/22 (32%) before, to 23/40 (58%) after bundle implementation. D-AED attachment occurred in 26/62 (42%), increase from 6/22 (27%) to 20/40 (50%). Cases with referral to a D-AED more often involved multiple bystanders onsite, 77% vs 23% in single bystander cases (*p* = 0.005). Median time difference between D-AED and EMS arrival was 03:14 (IQR 01:46–05:31) in the referral group and 01:49 (IQR 01:10–05:03) with non-referral (*p* = 0.8).

**Conclusion:**

Drones can deliver AEDs at an early stage in selected suspected OHCA cases. Implementation of an educational bundle at the dispatch centre was associated with increased dispatcher referral and bystander attachment of D-AEDs. D-AED referral was influenced by the number of bystanders onsite. When dispatcher referral occurred, D-AEDs were used in a majority of cases.

## Introduction

Despite widespread dissemination of automated external defibrillators (AEDs) in the community, mortality from out-of-hospital cardiac arrest (OHCA) remains as high as 90%.^1^, ^2^ Early defibrillation by bystanders using AEDs significantly increases the chance of survival.^3^, ^4^ However, publicly accessible AEDs are rarely within easy reach, and even when available, they are seldom referred to by the dispatch centre, retrieved by the bystander, or attached to the patient to potentially provide an early shock.^5^

In western Sweden, remotely operated, autonomously flying drones have previously been shown able to successfully deliver AEDs to patients in suspected OHCA, arriving before emergency medical services (EMS) in the majority (67%) of cases.^6^, ^7^ Drone delivery of AEDs (D-AED) has demonstrated a median time benefit of over three minutes compared to ambulance arrival — a time window sufficient for bystanders to retrieve and attach an AED,^8^ although these findings are specific to the Swedish context. In our previous study, D-AEDs were only attached and used in a minority (33%) of EMS-treated OHCA cases^7^ and although drone delivery of AEDs seem promising, survival from OHCA is heavily dependent on the referral from the dispatcher for bystanders to retrieve and use the D-AEDs.

Several barriers to emergency medical dispatch centres (EMDC) performance in initiating dispatcher-assisted CPR (DA-CPR) have been described, including agonal breathing, caller stress, language barriers, callers not being on scene, and inability to move the patient.^9^ There is, however, limited knowledge regarding EMDC performance during DA-CPR when D-AEDs are available, particularly with respect to dispatcher–bystander interaction during AED referral. This represents an important gap in current knowledge, as dispatcher communication and decision-making are central to successful implementation of AED-drone systems.

The aim of this study was to evaluate characteristics and changes over time regarding the interaction between dispatcher and bystander in referring bystanders to retrieve and use D-AEDs before and after implementing an educational bundle at the dispatch centre.

## Methods

### Study period and setting

This is a retrospective before–after observational study evaluating characteristics and barriers to dispatcher referral of bystanders to retrieve D-AEDs in suspected OHCA cases, as well as changes in referral and AED attachment rates following the implementation of a supportive and educational intervention bundle at the emergency medical dispatch centre.

Before the 33-month study period (01/06/2020–31/12/2023), AED-equipped drones were implemented at the EMDC and in EMS in western Sweden. Between June 1st and September 15th, 2020, three drones were operational, covering approximately 70,000 inhabitants. From April 21st, 2021, the same three drones were again operational with a fourth drone added on August 25th, 2021 and a fifth on November 23rd, 2021, expanding the total coverage area to approximately 200,000 inhabitants. Each drone operated within a 6 km radius from its hangar, all located in the Västra Götaland region of Sweden, please see Supplemental 1 for map and additional information. The study was approved by the Swedish ethics review authority registration no. 2019–06139 and 2020–06906.

### Drones, AEDs, and dispatch system

The drone systems were developed and operated by the drone operator Everdrone. DJI Matrice 600 Pro hexacopter drones equipped with Schiller FRED Easyport AEDs were used for the entire study period. Drones had a maximum velocity of 60 km/h and operated at an altitude of up to 65 m during flight. On arrival AEDs were winched down from an altitude of 30 m and generally delivered within 20 m from the location of the suspected OHCA. When a D-AED had been delivered, dispatchers were notified and could refer bystanders to retrieve it. Drones were restricted from flying in rain or when wind speeds exceeded 8 m/s. Operating hours were limited to the opening times of controlled airspaces and local air traffic control (ATC), typically between 08:00 and 22:00. A detailed description of the drone technology and dispatch system has been published previously.^6^, ^7^

### Bundle of interventions at the dispatch centre

For the first 2-year period of the feasibility study between June 2020 and May 2022 information on D-AED availability and modified telephone-assisted CPR guidelines was made available to dispatchers at the primary service answering point (PSAP) SOS Alarm (answering 112-calls) and at the regional EMDC in Västra Götaland region (SvLc). Dispatchers were told to refer callers to retrieve the D-AED if arrival occurred before EMS. An onsite study coordinator was available at the EMDC, reminding colleagues of the study procedures.

In June 2022, a specific educational and supportive bundle of interventions aiming to optimize dispatcher referral to D-AEDs was implemented, consisting of:

1. A 20-min E-learning program aimed at all dispatchers at the PSAP and registered nurses (RN) at the EMDC, with information on recognition of OHCA, Dispatcher assisted CPR and instructions on how to refer bystanders to retrieve D-AEDs.
2. An AED (training version) of the Schiller FRED Easyport AED with a basket identical to the real-life D-AED equipment was placed at the dispatch centre for “touch and feel” purposes.
3. Hands on – desktop information was available at each dispatcher workstation containing the instructions on what to tell the caller after drone delivery of an AED.
4. A Helicopter Emergency Medical Services-Coordinator (HEMS-C) at the EMDC supported the dispatcher verbally during emergency 112 suspected OHCA-calls, reminding them not to forget to refer the caller to the D-AED.
5. For HEMS-C assistance, an IOS software application was developed visualizing the drone on a map, en-route to the site on a tablet. On drone arrival at the site, a photo of where the D-AED was delivered on the ground was shown in the app on the tablet. This supported dispatchers during the 112-call in referring and guiding the bystanders to the exact spot where the D-AED was laying on the ground.^10^

In addition to this interventional bundle at the dispatch centre, media coverage and publications from previous studies led to increased awareness of the project in 2021 and 2022.^6^, ^8^

### Study eligibility criteria and terminology

All cases of suspected OHCA in which a D-AED arrived before EMS were included in this study. Exclusion criteria were EMS-witnessed arrests, missing emergency 112 audio recordings, and cases where EMDC aborted the drone mission due to new information in the 112-call after take-off, commonly non-OHCA cases. All cases in which CPR was initiated by a bystander were included, regardless of whether cardiac arrest was later confirmed by the EMS crew and reported to the Swedish Register for Cardiopulmonary Resuscitation. During the initial minutes of a 112 call, it is often unclear whether a true out-of-hospital cardiac arrest is present. According to guidelines, dispatchers should therefore encourage CPR on broad indications, such as unconsciousness and abnormal or absent breathing.^9^ This inclusion criterion reflects the real-time decision-making context at the emergency medical dispatch centre (EMDC), where dispatchers must act based on limited information. When CPR is deemed necessary during the emergency call, dispatchers initiate dispatcher-assisted CPR and, when feasible, refer the caller to retrieve a nearby AED, either publicly accessible or drone-delivered. In this study, the term “referral” indicated that the dispatcher, during the 112-call, gave a clear and explicit instruction to the caller to retrieve the D-AED after it had been delivered on site. Indirect mentions of the D-AED or non-directive prompts were not classified as referral. The term “retrieval” indicated that a bystander physically picked up the D-AED.

### Data sources

#### Dispatch centres

Data from the national (PSAP) dispatch centre SOS Alarm AB (CoordCom database) on time delays for fire department, EMS and drone units were collected. GPS coordinates were used to retrospectively determine arrival time of EMS units and to increase the precision of arrival onsite. Voice logs from emergency 112-calls were audited from both SOS Alarm AB and the regional EMDC, depending on which dispatch centre handled the emergency 112-call. All calls were audited by two of the study authors (SS and AC), and information on characteristics was gathered using a standardized template based on a modified version of the Cardiac Arrest Registry to Enhance Survival (CARES)^11^ (Supplemental 2). Every case was described in terms of whether the D-AED was mentioned during the 112- call and if a reason was found as to whether the dispatcher did or did not refer the bystander to retrieve the AED. Type of delivery site, residential or public, as well as time delays for EMS and barriers to DA-CPR were also included. Cases where the drone was deployed but did not deliver the AED or delivered it after EMS arrival excluded from audit.

#### Ambulance charts

Information on suspected OHCA cases was gathered from ambulance charts. These charts included data on characteristics, i.e., CPR, time delays and treatment during advanced life support (ALS), including defibrillation. Data was retrieved from three EMS services, cases of suspected OHCA were categorized depending on EMS-reports on whether CPR had been initiated or not.

Outcomes:

– Proportion (%) of cases where a dispatcher verbally (in the emergency call) referred a bystander to retrieve a D-AED (in total and before and after interventional bundle June 2022).
– Proportion of attached D-AEDs (% of cases with D-AED on scene).
– Proportion of defibrillated patients (% of cases with D-AED on scene)
– Characteristics of cases: referral/no referral (barriers to referral)
  o Single bystander (%)
  o Bystander with patient (%)
  o Distraught bystander (%)
  o Unwilling bystander (%)
  o EMS in immediate proximity (%)
  o Language barriers (%)
  o Call disrupted early (%)

### Statistical methods

Descriptive statistics are presented as counts and proportions. Associations between potential explanatory variables and dispatcher referral of bystanders to retrieve a D-AED, as well as D-AED attachment, were assessed using separate univariable logistic regression models. Results are reported as odds ratios (ORs) with 95% confidence intervals (CIs). Analyses were intentionally unadjusted, as the primary aim of the study was to explore factors associated with referral and attachment rather than to estimate independent causal effects. Although retrieval of a D-AED is required for attachment, not all retrieved AEDs were attached; therefore, attachment remained a variable outcome.

## Results

**Fig. 1:**
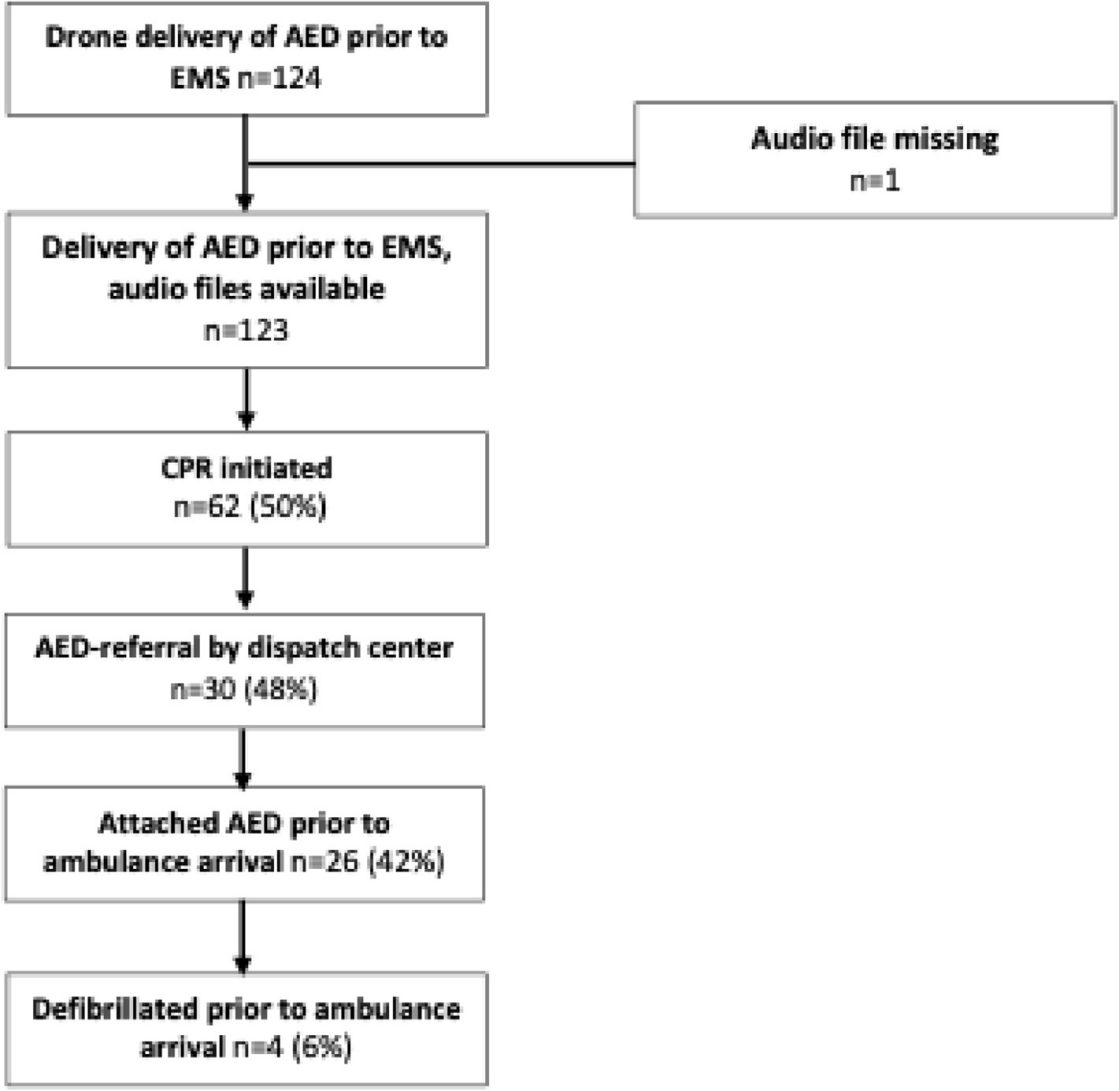
**Flow chart of D-AEDs in suspected OHCA**.

In total, *n* = 124 cases of AED deliveries by drones to suspected OHCAs occurred before arrival of EMS. In one case, the 112-audio file was unavailable, thus resulting in *n* = 123 cases included in further analysis (Fig. 1). Totally 123 cases of D-AEDs to suspected OHCAs occurred before EMS arrival over the study period. Out of these, 62/123 cases (50%) where considered as OHCA during the first minutes with initiation of bystander CPR, whereas 61/123 (50%) showed signs of life, had reluctant bystanders or apparent death.

**Fig. 2:**
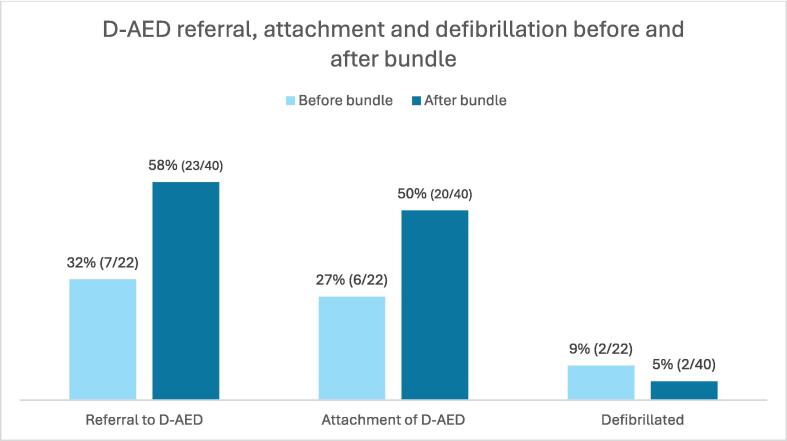
**D-AED referral, attachment and defibrillation before vs after bundle**. Referral to, attachment of, and defibrillated by D-AED before and after bundle implementation.

**Fig. 3:**
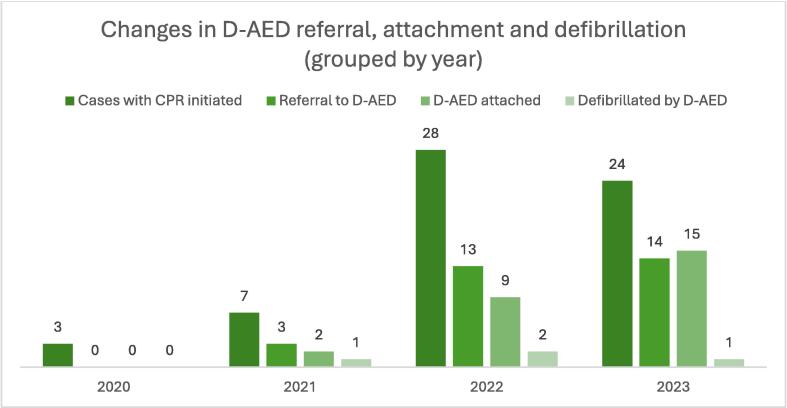
**D-AED referral, attachment and defibrillation by year.** Changes in referral, attachment, and defibrillation by D-AED s by year, absolute numbers. *D-AED = drone-delivered AED.

Referral, attachment and defibrillation before vs after bundle implementation Of all cases receiving bystander CPR, dispatcher referral to retrieve the D-AED occurred in totally 30/62 (48%) cases with an increase from 7/22 (32%) before the bundle implementation to 23/40 (58%) after. D-AED attachment occurred in 26/62 (42%) an increase from 6/22 (27%) to 20/40 (50%) after the bundle implementation. Totally 4/62 (6%) of patients were defibrillated, equally 2 cases before- and 2 after the intervention (please see Table 1, Table 2, Fig. 2, Fig. 3).

**Table 1:** Characteristics of D-AEDs and dispatcher referral.

All cases where CPR was initiated are included *n* = 62. Comparison between cases with dispatcher referral (*n* = 30) vs non-referral (*n* = 32). Odds ratios- OR with 95% confidence interval, and forest plot. A *p*-value of <0.05 was considered significant. Abbreviations: AED – automated external defibrillator, EMDC – emergency medical dispatch centre, EMS – emergency medical services, DA-CPR – dispatcher assisted cardiopulmonary resuscitation, OHCA – out-of-hospital cardiac arrest, MM:SS – minutes and seconds.

**Table 2:** Characteristics of D-AEDs and attachment of AEDs.

All cases where CPR was initiated are included *n* = 62. Comparison between cases with D-AED attached (*n* = 26) vs not attached (*n* = 36). Odds ratios- OR with 95% confidence interval, and forest plot. A *p*-value of <0.05 was considered significant. Abbreviations: AED – automated external defibrillator, EMDC – emergency medical dispatch centre, EMS – emergency medical services, DA-CPR – dispatcher assisted cardiopulmonary resuscitation, OHCA – out-of-hospital cardiac arrest, MM:SS – minutes and seconds.

The median time difference between D-AED and EMS arrival was 03:14 (IQR 01:46–05:31) in the referral group (30/62 cases) and 01:49 (IQR 01:10–05:03) in the non-referral group 32/62 cases), *p* = 0.8.

### Obstacles to referral in suspected OHCA

#### Factors concerning patient, caller and call

One factor for successful referral of bystanders to retrieve D-AED was multiple bystanders onsite. Out of all of cases with D-AED referral, *n* = 30, totally 77% occurred in cases with multiple bystanders onsite vs only 23% in single bystander cases (*p* = 0.005). Other reasons for non-referral were call disrupted early (16%), distraught caller (16%) unwilling caller (16%) and language barriers (9.4%) (please see Table 1, Table 2).

#### Factors concerning technicalities/response times

**Fig. 4:**
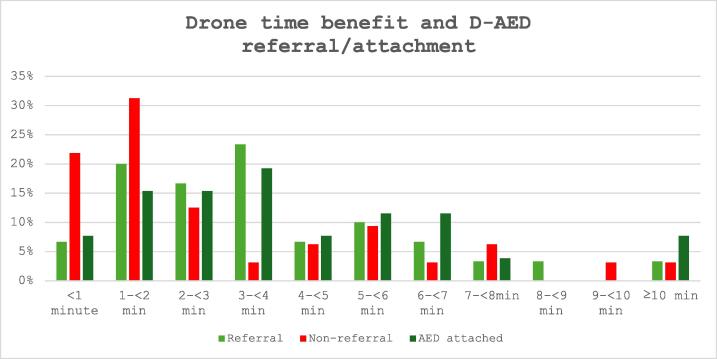
**Distribution of time benefit between D-AED arrival and EMS arrival**. Proportion of cases by time advantage of drone-delivered AED compared with EMS arrival, stratified by whether the D-AED was referred to, not referred to, or attached. The *x*-axis shows time advantage categories (<1 min, 1–<2 min, 2–<3 min, etc.), and the *y*-axis shows the proportion of cases within each category. Across increasing time advantage categories, the proportion of non-referral decreases, while referral and D-AED attachment become more common.

The median time difference when drones arrived prior to EMS in cases of suspected OHCA that received CPR was 01:49 (IQR 01:10–05:03) in the non-referral group and 03:14 (IQR 01:4–05:31) in the referral group. The same was seen in cases with D-AED attachment compared with cases without (Table 1, Table 2). Cases where the D-AED was referred to or attached more often had longer time advantages compared to EMS, whereas non-referred cases tended to have shorter time gains (Fig. 4).

## Discussion

### Main findings

In this study, we have, for the first time, described the interaction between callers and dispatchers at the EMDC when a D-AED is available for bystanders to retrieve. Our main finding is that the referral rate to D-AEDs increased from 32% to 58% after the introduction of an educational and supportive bundle of interventions at dispatch centres. Furthermore, we found several obstacles affecting dispatch personnel when referring callers to D-AEDs, including single caller, small time benefit compared with EMS, distraught/unwilling caller, and call disruption before instructions could be given (e.g. due to bad cellular connection leading to that the call is disrupted or that the caller hangs up the phone).

This study highlights the complexity of AED-drone systems. Previous studies on AED-drones have described potential time benefits compared with EMS or stationary AEDs,^12^, ^13^, ^14^, ^15^, ^16^, ^17^, ^18^, ^19^, ^20^ bystander perspectives^21^, ^22^ and most recently, real-life flights.^6^, ^7^, ^8^ However, the interaction between callers and dispatchers in real-life suspected OHCAs where a drone has delivered an AED has never been described before and the results of this study indicate that further interventions aimed towards both dispatch centres and the public are necessary to increase the use of D-AEDs.

### Associations between referral, retrieval, attachment and defibrillation

In most referral cases, D-AEDs were retrieved and often attached. However, some D-AEDs were not retrieved despite referral, while others were retrieved without referral—typically by volunteer responders alerted to the scene. Thus, referral does not guarantee AED retrieval or use. Additionally, even when a D-AED is attached before EMS arrival, defibrillation is only possible in patients with a shockable rhythm, which explains why not all attached AEDs resulted in shocks.

### Barriers to referral

Referral to a D-AED was less common when only one bystander was present, and dispatchers sometimes chose to prioritize continuous CPR over AED retrieval (23% vs 59%; OR 0.21, 95% CI 0.07–0.60). In the instructions to the dispatch centres, it was stated that in cases of multiple bystanders D-AED referral should always be done, whereas in the case of a single bystander, the dispatcher should assess the situation and decide from case to case on D-AED referral. It remains unclear whether uninterrupted CPR or early defibrillation offers greater benefit.

Although no statistically significant difference in median time advantage was observed between referral and non-referral cases, a small numerical difference of approximately 1.5 min was noted. This observation should be interpreted cautiously and does not provide statistical support for time advantage as a primary driver of referral. Rather, it suggests that perceived time benefit may play a contextual role in dispatcher decision-making. The dispatcher–caller interaction appears crucial; even a limited time advantage may result in AED retrieval when communication is clear and timely. This is illustrated by a case in which a D-AED was used despite an estimated time gain of approximately one minute.^8^ Drone placement significantly affects the chance of AED delivery before EMS and the potential time benefit.^23^

Distraught or unwilling callers were among the reasons found for non-referral in our study, consistent with prior findings identifying these as common barriers to CPR.^24^, ^25^ However, dispatchers can play a crucial role in overcoming this by encouraging emotionally stressed callers, potentially improving CPR initiation.^26^ Supporting dispatchers in managing such situations, alongside public CPR education, may enhance willingness to act and increase both CPR and AED use before EMS arrival.^27^

Language barriers were present in 9% of non-referral cases and none of the referral cases, suggesting they may hinder referral to D-AEDs. This aligns with previous findings that language barriers can delay treatment during emergency calls and DA-CPR.^28^, ^29^

Beyond the reasons already mentioned, non-referral was often unexplained, as dispatchers rarely verbalized their decisions during calls. A qualitative study (partly involving the same cases as in this study) found that D-AEDs posed challenges to protocol adherence. Dispatcher uncertainty, lack of routine clarity, and human factors likely contributed to non-referral. Technical and peer support were seen as facilitators, highlighting the need for targeted interventions at dispatch centres to improve referral rates.^30^

### AED availability

We observed a D-AED referral rate of 48% in cases of suspected OHCA where CPR was initiated—substantially higher than previous reports. Earlier Swedish and Danish studies showed referral rates of only 4% and 2.3%, respectively, despite AEDs being available within 100 m. A key difference is that D-AEDs in our study were typically placed within 10–30 m from the OHCA (rather than within 100 m), suggesting that proximity plays a crucial role.^5^, ^31^ Public AEDs are typically considered available if located within 100 m, based on AHA recommendations to retrieve an AED within 1.5 min.^32^ However, this guideline lacks strong evidence, and results from the two studies mentioned above show that AEDs within 100 m are rarely referred to or used.^5^, ^31^ In contrast, our study found a 48% referral rate with D-AEDs placed within 10–30 m, suggesting that a more realistic threshold for AED availability may be 30 m rather than 100.

## Limitations and methodological discussion

This is an observational study with many potential confounders affecting the understanding of the interaction between dispatchers and callers and the effects on intervention. Even though a standardized template was used when listening to emergency calls, the audit of 112-voice logs is subjective and does not necessarily reflect a clear understanding of the context and actions on-site. The exact reasons for non-referral or non-retrieval of D-AEDs are not always expressed by the dispatcher during the call. Furthermore, factors other than the educational intervention may have contributed to the observed increase in referral and D-AED attachment rates. These include increasing dispatcher experience with AED-drones over time, growing public awareness, and media coverage during the study period. Given the uncontrolled before–after design, secular trends and time-dependent learning effects cannot be fully disentangled from the effect of the educational bundle. All analyses were unadjusted, and the observed associations may therefore be influenced by confounding from unmeasured or correlated factors. As a result, the findings should be interpreted as descriptive associations rather than independent effects. Including all cases in which CPR was initiated, irrespective of later EMS-confirmed OHCA, may have diluted observed associations if non-arrest cases differed systematically in dispatcher behavior. However, this approach was chosen to reflect real-world dispatcher decision-making under time-critical conditions, where cardiac arrest cannot be reliably confirmed at the time of referral. The study was carried out in Sweden, and results may differ in other countries due to differences in EMS system structures, dispatcher training and protocols, drone availability and proximity, weather conditions, and local environmental factors such as distances and population density. No data on the experiences of bystanders were collected.

## Clinical implications

Although causal effects cannot be inferred, the findings of this study indicate that structured educational and supportive bundles at the dispatch centre may be a relevant component to consider when implementing or evaluating AED-drone programs.

## Conclusion

Drones can deliver AEDs at an early stage in selected suspected OHCA cases. Implementation of an educational bundle at dispatch centre was associated with increased dispatcher referral and bystander usage of D-AEDs. Dispatcher referral to D-AEDs were influenced by contextual factors, mainly the number of bystanders onsite. When dispatcher referral occurred, D-AEDs were used in a majority of cases.

## Declaration of generative AI and AI-assisted technologies in the manuscript preparation process

During the preparation of this work, the author used Microsoft Copilot to assist with language and phrasing. After using this tool, the author reviewed and edited the content as needed and takes full responsibility for the content of the published article.

## CRediT authorship contribution statement

**Sofia Schierbeck:** Writing – review & editing, Writing – original draft, Visualization, Project administration, Methodology, Investigation, Formal analysis, Data curation, Conceptualization. **Anette Nord:** Writing – review & editing, Supervision, Conceptualization. **Leif Svensson:** Writing – review & editing, Supervision, Methodology, Conceptualization. **Magnus Kristiansson:** Writing – review & editing. **Mattias Ringh:** Writing – review & editing, Methodology, Conceptualization. **Per Nordberg:** Writing – review & editing, Methodology, Conceptualization. **Jacob Hollenberg:** Writing – review & editing, Methodology, Conceptualization. **Gabriel Riva:** Writing – review & editing. **Martin Jonsson:** Writing – review & editing, Visualization, Formal analysis, Data curation. **Andreas Claesson:** Writing – review & editing, Supervision, Resources, Project administration, Methodology, Investigation, Funding acquisition, Conceptualization.

## Declaration of competing interest

The authors declare that they have no known competing financial interests or personal relationships that could have appeared to influence the work reported in this paper.
