# Supplementary material for: Dispatcher referral of bystanders to retrieve drone-delivered automated external defibrillators in cases of suspected out-of-hospital cardiac arrest
Source: Resusc Plus. 2026 Feb 16;28:101262. doi: 10.1016/j.resplu.2026.101262 (PMC12962124; doi:10.1016/j.resplu.2026.101262)
Supplement: Supplement 1 [file mmc1.docx]

# Supplemental 1 - Maps of drone areas

Maps of drone areas showing the drone hangar marked with a pin.

**Kungälv, Torslanda and Fiskebäck**


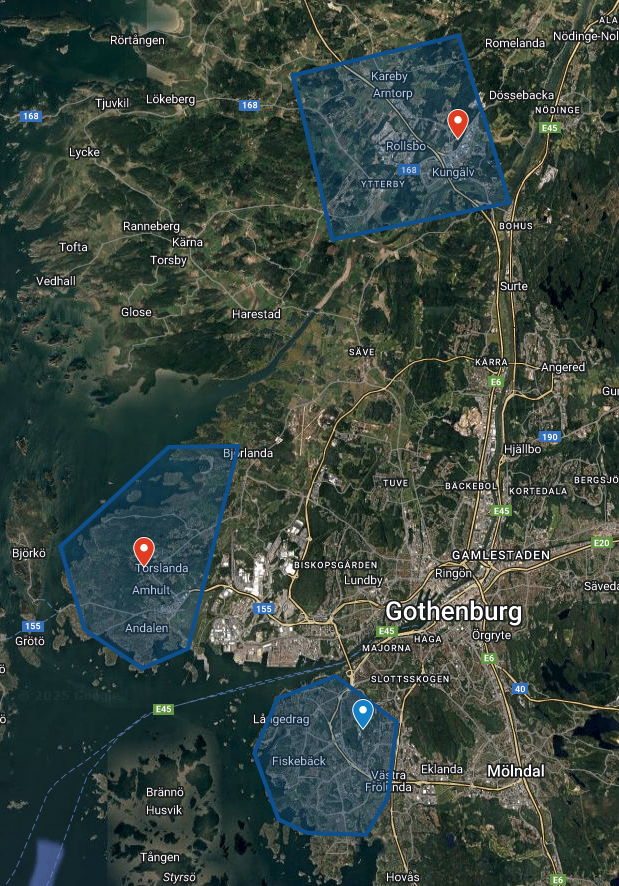


**Vänersborg and Trollhättan**

**
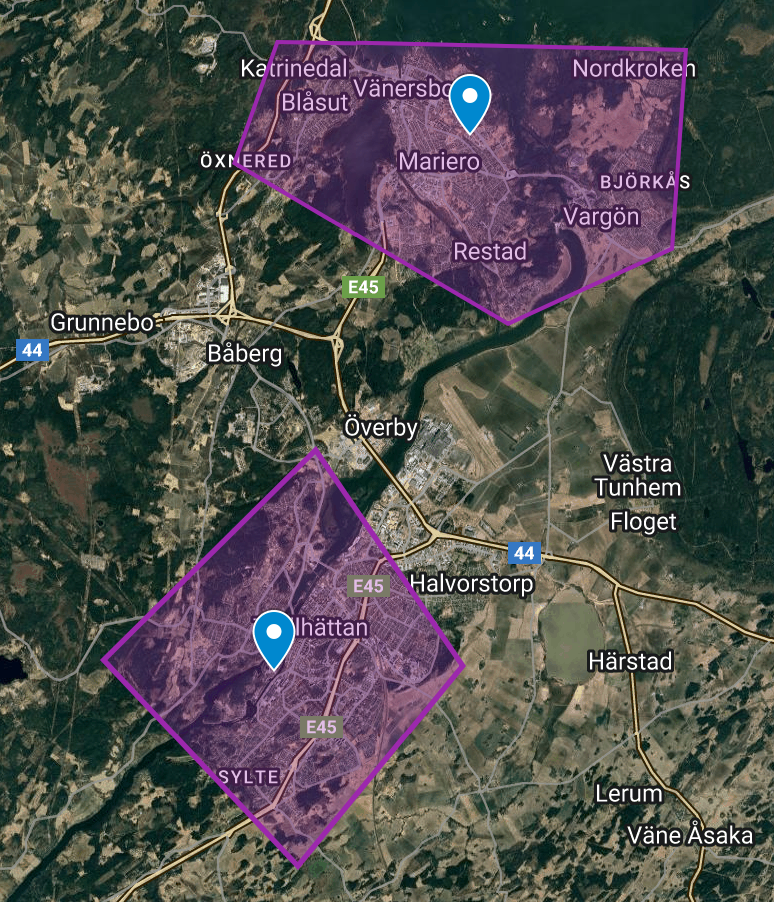
**
