# Supplementary material for: Dispatcher referral of bystanders to retrieve drone-delivered automated external defibrillators in cases of suspected out-of-hospital cardiac arrest
Source: Resusc Plus. 2026 Feb 16;28:101262. doi: 10.1016/j.resplu.2026.101262 (PMC12962124; doi:10.1016/j.resplu.2026.101262)
Supplement: Supplement 2 [file mmc2.docx]

# Supplemental 2 – modified CARES template

This template origins from AHA and The Cardiac Arrest Registry to Enhance Survival (CARES). It was used for audit of totally n=123 emergency 112-voice logs of all cases of drone delivered AEDs to suspected OHCA between 2020-2023. The template has been modified by adding variables on potential barriers for referring callers to retrieve drone delivered AEDs.

| Residential location? (1=residential, 0=public) |
| --- |
| If public (1=nature, 0=urban) |
| If residential (1=ground or first floor/outside, 0=floor 2 or more) |
| Number of bystanders (1=single, 0=multiple) |
| Bystander sex (1=man, 0=woman) |
| Caller with patient (1=yes, 0=no) |
| Relation to patient? (0=unknown/stranger, 1=family, 2=friend/acquaintance, 3=health care professional, 4=police) |
| Bystander age (0=child, 1=adult, 2=obviously elderly) |
| Does the dispatcher ask about consciousness? (1=yes, 0=no) |
| Does the dispatcher ask about breathing? (1=yes, 0=no) |
| Does the dispatcher ask about normal breathing? (1=yes, 0=no) |
| Does the patient breath normally? (1=yes, 0=no) |
| Is the need of CPR identified? (1=yes, 0=no) |
| Is the dispatcher active in giving instructions? (1=yes, 0=no) |
| Was CPR instructions given? (1=yes, 0=no) |
| Is CPR given? (1=yes, 0=no) |
| Type of CPR? (0=compressions only, 1=compressions + rescue breaths, 2=only rescue breaths) |
| Does someone on scene have CPR training? (1=yes, 0=no, 9=not asked) |
| AED is mentioned? (1=yes, 0=no) |
| Drone or drone-delivered AED is mentioned? (1=yes, 0=no) |
| Referral to the drone delivered AED is made? (1=yes, 0=no) |
| Drone-delivered AED is retrieved? (1=yes, 0=no) |
| Drone-delivered AED is attached? (1=yes, 0=no) |
| Other AED is retrieved? (1=yes, 0=no) |
| Other AED is attached? (1=yes, 0=no) |
| Defibrillation is given before EMS arrival? (1=yes, 0=no) |
| Language barrier? (1=yes, 0=no) |
| Distraught caller? (1=yes, 0=no) |
| Unwilling caller? (1=yes, 0=no) |
| Probable OHCA? (1=yes, 0=no) |
| Patient awake? (1=yes, 0=no) |
| Clear signs of death? (1=yes, 0=no) |
| EMS in immediate vicinity? (1=yes, 0=no) |
| Call disrupted early? (1=yes, 0=no) |
| Other comments |
